# Machine Learning Prediction of Transport Properties in Amorphous Polymer Electrolytes Using Chemically Informed Structural Descriptors

**DOI:** 10.1021/acsomega.6c09949

**Published:** 2026-10-22

**Authors:** Srinithin Ganesh, Tyler Paupst, Paromita Nath, Aditya Dilip Lele

**Affiliations:** † 5970 West Windsor-Plainsboro High School South, West Windsor, New Jersey 08550, United States; ‡ Department of Mechanical Engineering, 3536 Rowan University, Glassboro, New Jersey 08028, United States

## Abstract

Amorphous polymer electrolytes are promising candidates for next-generation lithium batteries. They offer improved safety and mechanical stability compared to conventional liquid electrolytes. However, predicting their transport performance remains challenging. Ionic conductivity and diffusivity depend on complex, coupled interactions between polymer structure, chemistry, and ion dynamics. This work leverages machine learning to predict four key transport properties in amorphous polymer electrolytes using a molecular dynamics data set of more than 6,000 polymer electrolyte systems. These are ionic conductivity, bis­(trifluoromethanesulfonyl)­imide (TFSI) diffusivity, lithium-ion diffusivity, and polymer diffusivity. To enhance predictive capability and preserve mechanistic interpretability, we engineered chemically and structurally meaningful features from Simplified Molecular-Input Line-Entry System (SMILES) representations. Selected features include elemental composition, functional group densities, polarity descriptors, and polymer flexibility metrics. Three modeling frameworks were trained and evaluated under 5-fold cross-validation, including random forest, gradient boosting, and neural networks. The neural network achieved the highest predictive performance across all four targets and exceeded prior benchmarks on three of them. It also exhibited substantially greater robustness to data partitioning than the tree-based baselines. Feature importance analysis was performed using both impurity-based rankings and Shapley additive explanations. Polymer backbone flexibility, polarity, and ionic environment were found to dominate transport behavior. Directional attribution further identified primary and secondary amines and carboxylate groups as specific negative contributors to lithium-ion mobility. The results of this work demonstrate that chemically informed structural descriptors can effectively predict electrolyte performance while yielding mechanistic insight. This enables accelerated screening and design of high-efficiency polymer electrolytes for future energy storage applications.

## Graphical abstract

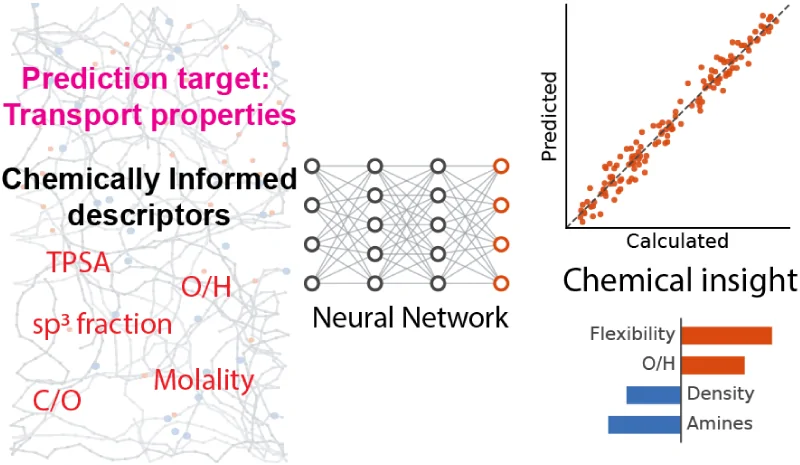

## 1 Introduction

Rechargeable lithium-ion batteries are central to modern energy storage, powering applications ranging from portable electronics to electric vehicles.^1,2^ Continued improvements in battery performance depend heavily on advances in electrolytes.^3–8^ Electrolytes mediate ion transport between electrodes during charging and discharging. While conventional liquid electrolytes provide high ionic transport, they also introduce safety challenges such as leakage and flammability, motivating strong interest in alternative electrolyte systems.^2,9^ Polymer electrolytes are a promising solution because they can offer improved mechanical stability, reduced leakage risk, and potential compatibility with next-generation battery chemistries, including lithium metal anodes.^9–12^ Despite this potential, designing high-performing polymer electrolytes remains challenging. The key transport properties that determine electrolyte performance, ionic conductivity and species diffusivities, depend on factors including polymer structure, chain flexibility, polarity, and salt concentration.^11,13^ In amorphous polymer electrolytes, these relationships are especially complex because ion movement is strongly coupled to local polymer motion and microscopic structural effects.^13,14^ As a result, evaluating candidate polymer electrolytes through experiments or detailed simulations can be time- and resource-intensive. Even when simulation data is available, extracting clear design rules is difficult as small structural changes can lead to large changes in transport behavior.

Machine learning (ML) offers a data-driven route to structure–property relationships that would otherwise require large numbers of experiments or detailed simulations. In broader materials chemistry, ML has successfully captured complex nonlinear relationships between molecular structure and thermophysical properties.^15–20^ These data-based techniques have been extensively used for polymer property predictions as well.^21–23^ In the context of polymer electrolytes specifically, ML models have been applied to predict key transport and electrochemical properties from molecular structure descriptors.^21^ Early data-driven approaches applied algorithms such as random forest, XGBoost, and kernel ridge regression.^24^ While effective for specific systems, these methods often fail to generalize across chemically diverse polymer families because simplified string representations cannot capture the chain-level topology and segmental dynamics that govern ion transport.^25,26^ To overcome these limitations, graph neural network (GNN) architectures have been increasingly adopted, wherein polymer structures are encoded as molecular graphs and task-relevant features are learned directly from atoms and bonds.^27^ A particularly notable development is the use of chemistry-informed neural networks that embed the Arrhenius equation directly into the model readout layer, enabling accurate prediction of temperature-dependent ionic conductivity across a wide range of solid polymer electrolyte formulations.^25^ Similarly, multitask GNNs trained on molecular dynamics (MD) simulation data have achieved state-of-the-art predictions of both conductivity and ion diffusivity, outperforming fingerprint-based baselines and generalizing well to experimental benchmarks.^28^ These studies highlight the ability of ML models to uncover nonlinear relationships in chemically complex systems, motivating its application to polymer electrolytes.

Hatakeyama-Sato et al.^29^ constructed a database of 240 lithium-ion conducting polymer electrolytes and applied gradient boosting models to predict ionic conductivity. Their work demonstrated that even relatively small data sets could yield meaningful predictions when appropriate descriptors were selected. Importantly, they identified monomer electronegativity and polarity as dominant contributors to conductivity, emphasizing the importance of chemically meaningful features. However, the relatively small data set (240 systems) limited the accessible polymer chemical space and model generalization. This motivates the present work, which leverages a substantially larger MD data set containing 6,270 polymer electrolyte systems to capture broader structural diversity and improve prediction of multiple transport properties. More recently, hierarchical polymer graph representations paired with graph attention networks have demonstrated improved accuracy and generalization for structurally complex systems including block copolymers and branched architectures.^27^ Bradford et al.^25^ developed a chemistry-informed neural network (ChemArr) trained on over 7,000 experimentally reported ionic conductivity values. By embedding the Arrhenius equation directly into the model architecture, they incorporated physical constraints into a message passing neural network framework. This chemistry-informed approach improved predictive accuracy and generalization compared to purely data-driven models. Their study demonstrated the power of integrating physical laws with deep learning but focused primarily on predicting ionic conductivity rather than diffusivity components. More recently, Han et al. introduced a hierarchical polymer graph (HPG) representation combined with graph attention networks (GATs) to predict ionic conductivity across diverse polymer architectures, including block and branched systems.^27^ Their hierarchical representation addressed limitations of conventional SMILES-based descriptors by capturing both monomer-level and polymer chain-level structural information. While this deep learning approach improved generalization and captured temperature-dependent behavior, the work primarily emphasized representation learning rather than detailed feature interpretability or comparison with ensemble tree-based methods.

Considering these investigations, several gaps remain in using data-based approaches for polymer electrolyte property predictions. First, most existing studies target a single transport property, most often ionic conductivity, leaving diffusivity components comparatively underexplored despite their mechanistic importance for ion transport. Second, although some models incorporate physics-informed constraints, many do not provide clear interpretability regarding which structural descriptors most strongly drive performance. Third, while deep learning models often achieve high predictive performance, there is limited analysis of whether their added complexity yields tangible benefits over ensemble tree-based methods when both are trained on identical feature sets, particularly when interpretability is weighed against predictive gains. The primary objective of this work is therefore to train and compare ML models that predict multiple polymer electrolyte properties using interpretable structural descriptors.

This objective is accomplished by applying machine learning to predict four key transport targets in amorphous polymer electrolytes: ionic conductivity, bis­(trifluoromethanesulfonyl)­imide (TFSI) diffusivity, lithium-ion diffusivity, and polymer diffusivity. Selected models are trained using a high-throughput MD data set of 6,270 trajectories reported by Xie et al.^14,30^ While Xie et al.^14,30^ previously benchmarked random forest and graph neural network models on this data set, their study emphasized standard model architecture performance without considering engineered chemical descriptors or feature interpretability. Their reported R^2^ values provide a baseline for comparison in the present work and are quoted alongside our own results in Section 3.1. Building upon this foundation, we engineer chemically and structurally meaningful features derived from SMILES representations and evaluate three model families: random forest, gradient boosting, and neural networks. These models were selected to span bagged ensembles, boosted ensembles, and neural architectures, balancing interpretable tree-based approaches against a more flexible nonlinear model on the same engineered feature set. Beyond predictive performance, we emphasize feature importance analysis to identify which structural descriptors most strongly influence transport behavior. By systematically comparing ensemble and neural architectures while maintaining interpretability, this study provides both predictive insight and practical guidance for future electrolyte design. This work is organized as follows. Section 2 details the data set, feature engineering, ML methods, and evaluation criteria used in this study. Section 3 presents and discusses the predictive performance of the three model families, benchmarks the results against prior work, and examines feature importance to identify the structural descriptors that most strongly influence transport behavior. Section 4summarizes the key findings of this work.

## 2 Materials and Methods

This section outlines the data sources, feature engineering strategies, modeling frameworks, and evaluation procedures used to predict key transport properties in amorphous polymer electrolytes (see Figure 1). We first describe the MD-derived data set used in this work and the subsequent feature construction pipeline. Next, we detail the rationale behind selected features and their physicochemical relevance to ion transport. Model development is then presented across three machine learning algorithms: random forest, gradient boosting, and neural networks. The training protocols and data set partitioning approaches for each algorithm are also described in detail. Finally, we define the performance metrics used to assess predictive performance and describe the feature attribution methods used to interpret each model.

**Figure 1:**
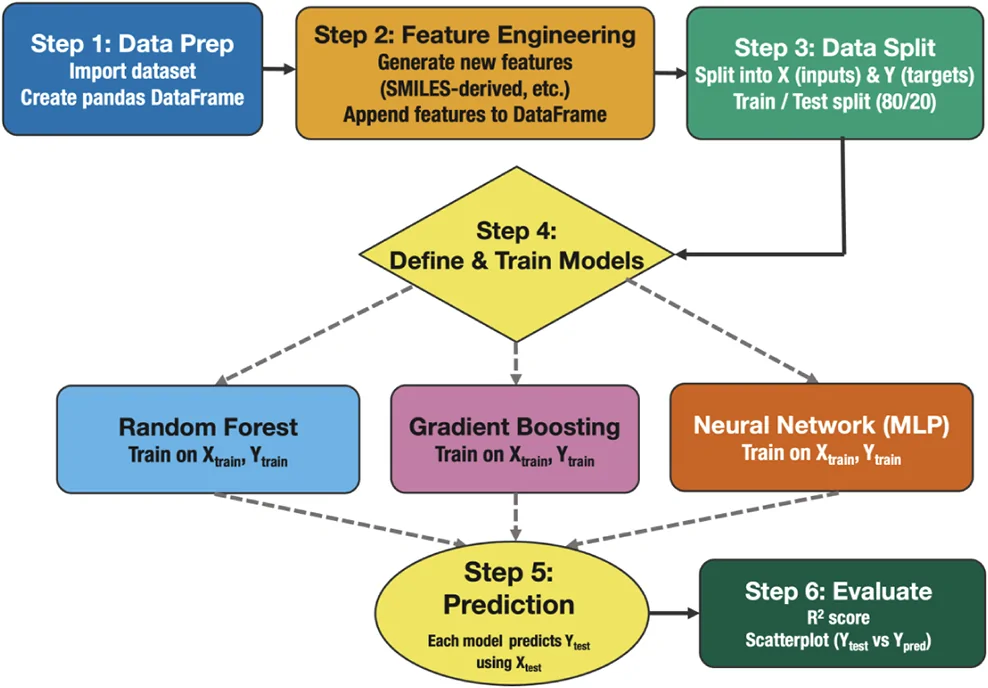
Overview of the machine learning workflow for predicting transport properties in amorphous polymer electrolytes. The pipeline includes data preparation and feature engineering from SMILES-derived descriptors, an 80/20 train–test split, model training using random forest , gradient boosting, and neural network architectures, followed by prediction and performance evaluation using the R^2^ score and predicted-versus-actual scatter plots.

### 2.1 Data Set

The data set used in this work was obtained from the work of Xie et al.,^30^ which presents a cloud-based platform designed for sharing and automated analysis of raw data from high-throughput polymer MD simulations. This platform houses a substantial data set comprising 6,270 MD trajectories of amorphous polymer electrolytes. The initial 3D structures of the polymer electrolytes were constructed by incorporating 1.5 mol of lithium bis­(trifluoromethanesulfonyl)­imide (LiTFSI) per kilogram of polymer, corresponding to 50 LiTFSI ion pairs. These ions were inserted into a mixture of polymer chains, and the system was equilibrated using 5 ns MD simulations at 353 K. This procedure generated realistic polymer-salt configurations for subsequent feature extraction and analysis. Model evaluation in this work used 5-fold cross-validation on the data set described earlier, with each fold using an 80/20 train-test partition of the data set. Details of the cross-validation protocol are given in Section 2.4. This data set split provides sufficient training data while reserving a subset for unbiased evaluation on unseen data. For polymer diffusivity, the removal of a small number of extreme outlier data points using manual inspection (with orders of magnitude difference as compared to the rest of the data set) was applied uniformly across all three models to ensure consistent evaluation conditions. This filtering reduced the data set from 6,270 to 6,253 samples and improved stability across model families. No additional data set filtering was applied selectively to any single model. Differences in performance therefore arise from model architecture and hyperparameter choices rather than from inconsistencies in training data.

### 2.2 Features

The data set^30^ includes MD simulation geometry-derived features such as molality, monomer molecular weight, degree of polymerization, density, and SMILES representations of the polymers. The data set also contains our target features:^30^ Conductivity (ionic charge transport), TFSI diffusivity (anion mobility), Li-ion diffusivity (cation mobility), and polymer diffusivity (polymer chain segment motion). These properties were selected as target variables because they directly affect ion transport behavior and overall electrical performance in polymer electrolytes. Accurately predicting conductivity and diffusivities enables rapid screening of candidate materials for high-efficiency lithium battery applications.

To enhance the predictive power of the data set, additional training features (see Table 1) were engineered from the SMILES representations of the polymer electrolytes. Features were selected a priori based on established physicochemical relationships with ion transport in polymer electrolytes,^31–34^ ensuring that every descriptor carries a clear mechanistic interpretation. This hypothesis-driven approach was chosen over automated feature selection to preserve interpretability of the resulting feature importance rankings, which would otherwise be confounded by data-driven pruning. Elemental counts (C, O, N, F, H, S, Cl, Si, P) reflect overall composition and polarity, influencing dielectric constant, coordination strength, and how well lithium salts dissolve and dissociate, which are key factors for conductivity and ion diffusivity.^13,35,36^ Polymer-relevant ionic site counts (carboxyl/carboxylate and primary/secondary amines) represent binding or coordination sites that modulate ion dissociation, association, and mobility. Polar groups can anchor or solvate Li^+^, either promoting ion motion by stabilizing charges or hindering it when binding is too strong.^37^ Topological and molecular descriptors such as Topological polar surface area (TPSA), FractionCSP3, and number of rotatable bonds relate to polymer polarity, conformational flexibility, and backbone dynamics, all of which correlate with segmental motion and the ease with which ions travel through the material.^13,38,39^ Composite features, including C/O and O/H ratios, fluorine fraction, monomer size per repeat unit, and ionic density, serve as proxies for electronegativity gradients, chain architecture, and local charge concentration. These composite features also capture the coupling between polymer segmental motion and ionic transport.^13^ Finally, atom density is included as a free-volume proxy, since looser chain packing has been shown to improve ionic conductivity by reducing steric hindrance to ion motion.^12^ Together, these features integrate compositional, structural, and dynamic signals known to influence conductivity and the diffusivities of TFSI, Li^+^, and the polymer matrix itself. Table 1 lists all the input and target features used in this work.

**Table 1:** Summary of Input Features (X) and Target Properties (Y) Used for Machine Learning Model Development

**Input Features(X)**			**Target Features(Y)**
Molality	# of Fluorine	Carboxyl or Carboxylate	Conductivity
Monomer Molecular Weight	# of Hydrogen	Primary or Secondary Amine	TFSI Diffusivity
Density	# of Sulfur	Topological Polar Surface Area (TPSA)	Polymer Diffusivity
# of Carbon	# of Chlorine	Carbon Saturation Ratio (FractionCSP3)	Li-Ion Diffusivity
# of Oxygen	# of Silicon	Number of Rotatable Bonds	
# of Nitrogen	# of Phosphorus	C/O Ratio	
Atom Density	Fluorine Fraction	O/H Ratio	
Monomer Size per Repeat Unit	Ionic Density		

### 2.3 Models

Three types of ML models are evaluated in this study: random forest,^40^ gradient boosting,^41^ and neural networks,^42^ to compare interpretable tree-based baselines against a more flexible nonlinear model on the same engineered feature set. The specific hyperparameter ranges explored, the outcome of the tuning procedure for each model, and the final configuration values are discussed in detail in Section 3.2.

#### 2.3.1 Random Forest

Random forest is an ensemble machine learning algorithm that constructs a collection of decision trees during training and outputs the average prediction (for regression) or majority vote (for classification) of these trees. Each tree is trained on a bootstrap sample^40^ of the original data set, introducing variability, while at each split, only a random subset of features is considered. This randomness reduces model variance and mitigates overfitting, improving generalization compared to a single decision tree. By aggregating predictions across multiple weak learners, a random forest approach captures nonlinear relationships and complex feature interactions effectively, making it particularly suitable for high-dimensional data sets with interdependent features. Random forest was selected for this study as the aforementioned strengths make it well positioned for the complex characteristics of polymer electrolyte systems. The ensemble approach facilitates the capture of intricate relationships between structural, chemical, and thermodynamic descriptors. The random forest regressor was implemented using the SciKit-Learn library.^43^ The model was then trained using the data set described in Section 2.1 and used to predict the target features from electrolytes in our test data set. The final model’s hyperparameters included: 800 estimators, a maximum tree depth of 20, minimum samples per leaf of 5, minimum samples per split of 10, and parallel processing enabled through *n_jobs = −1*, with a fixed *random_state = 42* to ensure reproducibility. These configuration values were selected through the tuning process described in Section 3.2.

#### 2.3.2 Gradient Boosting

A gradient boosting regressor^41^ was implemented using the SciKit-Learn library^43^ to evaluate the effectiveness of sequential ensemble learning for property prediction. Unlike random forest, which aggregates predictions from independently trained trees, gradient boosting constructs trees iteratively, where each successive tree corrects the residual errors of the previous ensemble to progressively minimize bias. This boosting strategy enables the model to learn complex nonlinear relationships by focusing learning capacity on difficult-to-predict regions of the feature space. In gradient boosting, each tree is trained on the negative gradient of the loss function with respect to the current model predictions, effectively performing functional gradient descent in model space. For regression tasks such as this work, this typically corresponds to minimizing mean squared error, meaning each new tree is optimized to reduce the remaining prediction error of the ensemble. This sequential error-correction process allows gradient boosting to achieve lower bias than bagging-based methods, while carefully applied regularization controls variance.^41^ Consequently, gradient boosting often performs well on structured scientific data sets where subtle feature interactions influence target transport or thermodynamic properties. Similar to random forest, the model was trained using our training data set and evaluated on an independent testing sample to assess generalization performance. The hyperparameters of the final model were set to: 500 estimators, a learning rate of 0.1, a maximum tree depth of 3, *min_samples_split = 10*, *min_samples_leaf = 5*, and *max_features = 0.7*, with a fixed *random_state = 42* to ensure reproducibility. These configuration values were selected through the tuning process described in Section 3.2.

#### 2.3.3 Neural Network

A neural network^42^ is a machine learning model inspired by the structure and function of the human brain. It is composed of interconnected layers of artificial neurons. Each neuron receives input signals, applies a weighted transformation, and passes the result through a nonlinear activation function to the next layer. By stacking multiple layers, including input, hidden, and output layers, neural networks can learn complex nonlinear relationships between input features and target outputs. Through a process called backpropagation, the network iteratively adjusts its weights to minimize the difference between predicted and actual values. This enables the capture of intricate patterns and interactions within high-dimensional data sets. Neural networks are particularly effective when relationships between features and targets are highly nonlinear.^42^ The implemented neural network is a feedforward, fully connected architecture (multilayer perceptron, MLP) through PyTorch.^44^ The neural network is used to predict target properties from polymer electrolyte features. The input features are first standardized using *StandardScaler*. Standardization was applied only for the neural network because gradient-based optimization is sensitive to input scale, whereas random forest and gradient boosting are invariant to monotonic transformations of individual features. The data set is split into training and testing sets as described in Section 2.1 and converted into PyTorch^44^ tensors for computation. The MLP architecture consists of an input layer matching the number of features, followed by two hidden layers of 128 neurons each with ReLU activation functions, and a single linear output neuron for regression. The network is trained using the Adam optimizer^45^ with a learning rate of 0.001 and mean squared error (MSE) as the loss function.

Training of the neural network utilized mini-batch gradient descent with early stopping. The training data was partitioned into small batches using PyTorch’s^44^ DataLoader, allowing the model to update weights more frequently per epoch. This improves convergence stability and reduces the risk of stagnation due to local minima. The batch size was selected empirically by evaluating multiple candidate values and comparing validation loss and predictive performance. A batch size of 16 produced the most stable convergence and lowest validation error across most targets. Early stopping was employed to prevent unnecessary training beyond reasonable performance increase. The validation loss was monitored at each epoch and training was terminated if no improvement was observed for 20 consecutive epochs. The best model parameters were saved for subsequent evaluation.^42^ This combination of mini-batch training and early stopping ensured efficient learning while reducing overfitting. This is particularly important for the relatively small and high-dimensional polymer electrolyte data set.

### 2.4 Evaluation Metrics and Analysis

To assess robustness of predictive performance across data partitions, the investigated models were evaluated using 5-fold cross-validation with shuffled splits (*random_state* = 42). The same fold indices were used across models within each target to enable direct fold-level comparison of architectures. For the neural network, feature and target *StandardScaler* transformations were fit on training folds only to prevent information leakage. Model performance was evaluated using the coefficient of determination (R^2^ score) as a standard metric. R^2^ quantifies the proportion of variance in the target variable that is explained by the model.^46^ An R^2^ value of 1 indicates perfect prediction, while a value of 0 suggests that the model performs no better than simply predicting the mean of the target. This metric is particularly useful because it provides a normalized measure of predictive accuracy across different target scales, allowing direct comparison between models or features. As such, test-set R^2^ values are reported as mean ± standard deviation across the five folds. Because the transport targets span several orders of magnitude, we additionally report the mean absolute error of log_10_ transformed predictions (*log*_10_ *MAE*), defined as

$$log_{10}\;MAE=\left(1/N\right)\Sigma_{i}\left|{log}_{10}\left({ŷ}_{i}\right)-{log}_{10}\left(y_{i}\right)\right|$$

where ŷ*_i_* and *y_i_* are the predicted and MD reference values. This metric quantifies typical relative error; a *log*_10_ *MAE* of 0.1 corresponds to a typical deviation of a factor of 10^0.1^ ≈ 1.26 from the MD value. Because it depends only on the ratio of predicted to reference values, it is unaffected by the target rescaling described in Section 3.3. *log*_10_ *MAE* was computed on test-fold predictions from models trained on linear-scale targets. Nonpositive predictions, which occurred for fewer than 0.1% of test points, were excluded from this calculation.

In the context of polymer electrolyte modeling, R^2^ enables assessment of how well the model captures the relationships between chemical and structural descriptors and properties such as conductivity and diffusivity. To interpret model behavior and identify which input variables most strongly influenced predictions, feature importance analyses were conducted for all three architectures. The tree-based ensemble methods, random forest and gradient boosting, inherently compute importance scores based on how much each feature reduces prediction error across decision splits; these values were extracted through the built-in *feature_importances* attribute in Scikit-Learn.^43^ For the neural network, comparable attribution requires a posthoc technique, since neural networks do not expose intrinsic importance measures. To rectify this, we utilized Shapley additive explanations (SHAP),^47^ a game-theoretic framework that assigns each feature a contribution to every individual prediction by averaging its marginal effect across all possible feature subsets. SHAP values were computed on the held-out test set using the incumbent *DeepExplainer* implementation with a background sample of 100 training instances. The SHAP values were rescaled from the standardized training space back to physical target units so that reported contributions are directly interpretable in Scm^–1^ for conductivity and m^2^s^–1^ for diffusivities. SHAP provides two capabilities beyond impurity-based importance that are relevant to the present analysis: directionality, in that positive and negative contributions are distinguished, and per-sample attribution, which enables inspection of how feature effects vary across individual electrolyte systems.

## 3 Results and Discussion

The predictive performance of three machine learning architectures was evaluated for four transport targets in amorphous polymer electrolytes: ionic conductivity, TFSI diffusivity, Li-ion diffusivity, and polymer diffusivity. The three architectures were random forest, gradient boosting, and a neural network with early stopping. Model performance was assessed using the coefficient of determination (R^2^) under 5-fold cross-validation as described in Section 2.4, with per-fold and mean ± standard deviation values reported. Predicted-versus-actual plots were generated for each target and model combination from the same representative fold, specifically fold 1 of the 5-fold cross-validation. The same fold indices were used across all three architectures within each target, so the plots reflect predictions on an identical set of test polymers for each mode. For all three models, feature importance rankings were extracted to provide an interpretable link between the engineered chemical descriptors and the learned structure–property relationships. This combination allows both cross-model comparison of which descriptors matter most and, through the SHAP attribution, directional interpretation of how each descriptor influences transport. Section 3.1 compares overall this works’ model performances across targets against a benchmark from prior work on the same MD data set. Section 3.2 then examines per-model behavior and hyperparameter sensitivity, while Section 3.3 investigates the numerical scaling required to stabilize training on small-magnitude diffusivity targets. Finally, Section 3.4 discusses the use of feature importance analysis to interpret the dominant physicochemical mechanisms governing transport and discuss the associated data set limitations and implications for future ML frameworks.

### 3.1 Overall Model Performance and Comparison to Prior Work

Table 2 summarizes the cross-validated test-set R^2^ and *log*_10_ *MAE* values for all four transport properties across the three model architectures. The neural network achieved the highest mean R^2^ across every target, with values of 0.75 ± 0.12 for ionic conductivity, 0.75 ± 0.05 for TFSI diffusivity, 0.66 ± 0.07 for Li-ion diffusivity, and 0.78 ± 0.02 for polymer diffusivity. The random forest and gradient boosting baselines produced lower means with substantially greater fold-to-fold variability, particularly for conductivity and Li-ion diffusivity. Predicted-versus-actual comparisons for each model, generated from a representative fold, are shown in Figure 2, where deviations from the y = x line reflect model bias and variance for each target. Beyond the differences in mean performance, the cross-validation reveals a robustness gap that the single-split evaluation did not expose. For ionic conductivity, the neural network R^2^ ranged from 0.58 to 0.88 across all five folds, while the random forest R^2^ ranged from 0.13 to 0.77 and gradient boosting from 0.27 to 0.82. A similar but less extreme pattern was observed for TFSI diffusivity (neural network range 0.69 to 0.81 versus random forest range 0.34 to 0.76) and Li-ion diffusivity (neural network range 0.58 to 0.74 versus random forest range 0.40 to 0.70). The origin of the low-performing folds for the ensemble models is examined in detail in Section 3.2. A similar but less extreme pattern was observed for TFSI diffusivity (neural network range 0.69–0.81 versus random forest range 0.34–0.76) and Li-ion diffusivity (neural network range 0.58–0.74 versus random forest range 0.40–0.70). Polymer diffusivity was uniformly stable across folds and models, with all standard deviations at or below 0.02. This suggests that polymer chain motion is captured by a small number of consistently informative descriptors, whereas ionic conductivity and lithium mobility depend on descriptor combinations whose predictive value is more sensitive to which chemistries dominate the training set. *log*_10_ *MAE* values range from 0.100 to 0.135 across all models and targets, corresponding to typical prediction errors of 26 to 36% relative to the MD reference values. The neural network achieved the lowest *log*_10_ *MAE* on every target, although differences between architectures did not exceed 0.006. Because R^2^ is computed on a linear scale, it is dominated by systems with the largest transport values, whereas *log*_10_ *MAE* weights relative errors equally across the full range. The large spread in R^2^ alongside the small spread in *log*_10_ *MAE* therefore indicates that the architectures differ mainly in their predictions for high-transport systems.

**Table 2:** Comparative 5-Fold Cross-Validated R^2^ and *log*_10_ *MAE* (Mean ± Standard Deviation) of Random Forest, Gradient Boosting, and Neural Network Models across Key Transport Targets in Amorphous Polymer Electrolytes^a^

**Target**	**Metric**	**Random Forest**	**Gradient Boosting**	**Neural Network**	**Random Forest (**Xie et al.^14,30^ **) R** ^**2**^	**Neural Network (**Xie et al.^14,30^ **) R** ^**2**^
**Conductivity**	R^2^	0.60 ± 0.27	0.61 ± 0.23	0.75 ± 0.12	0.532	0.573
	*log*_10_ *MAE*	0.135 ± 0.002	0.132 ± 0.002	0.129 ± 0.002		
**TFSI Diffusivity**	R^2^	0.64 ± 0.17	0.68 ± 0.10	0.75 ± 0.05	0.633	0.650
	*log*_10_ *MAE*	0.116 ± 0.003	0.114 ± 0.002	0.111 ± 0.003		
**Li-Ion Diffusivity**	R^2^	0.56 ± 0.12	0.47 ± 0.14	0.66 ± 0.07	0.492	0.508
	*log*_10_ *MAE*	0.131 ± 0.003	0.129 ± 0.003	0.128 ± 0.005		
**Polymer Diffusivity**	R^2^	0.73 ± 0.02	0.74 ± 0.01	0.78 ± 0.02	0.820	0.832
	*log*_10_ *MAE*	0.105 ± 0.002	0.102 ± 0.003	0.100 ± 0.003		

a For Xie et al.,^14,30^ only R^2^ values are reported from single-split evaluations and are shown for reference.

**Figure 2:**
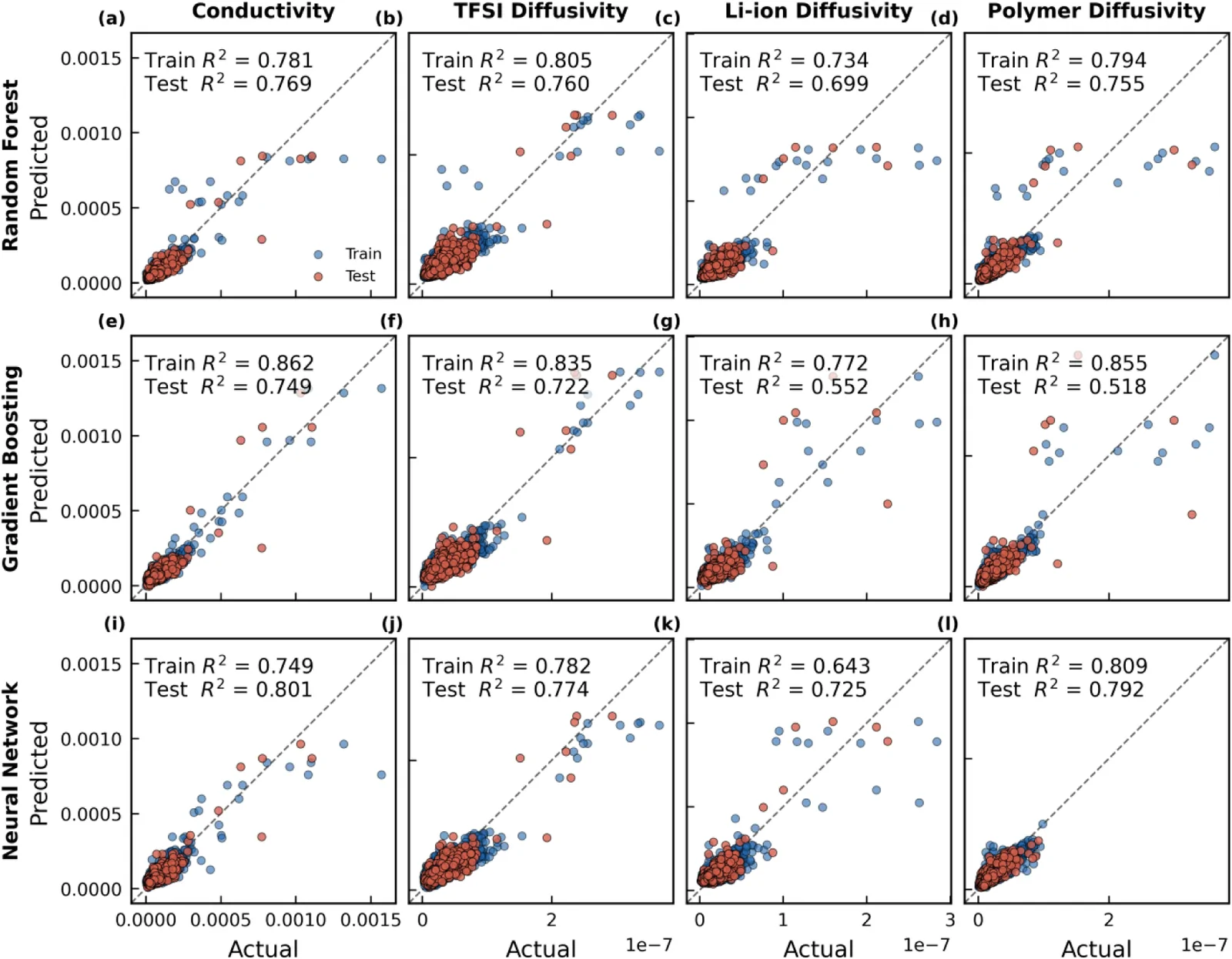
Predicted-versus-actual comparisons for ionic conductivity, TFSI diffusivity, Li-ion diffusivity, and polymer diffusivity across the three model architectures (random forest, gradient boosting, and neural network). Values shown are from a representative 80/20 train-test split (random_state = 42) corresponding to the first fold of the 5-fold cross-validation reported in Table 2. Deviations from the y = x line reflect model bias and variance for each target. Training and testing data are shown in blue and red circles respectively.

To compare the results to prior work, we consider the work of Xie et al.^14,30^ as a benchmark. For the neural network, our cross-validated results exceed the reported values of Xie et al.^14,30^ by approximately 0.10 to 0.18 in R^2^ for conductivity, TFSI diffusivity, and Li-ion diffusivity. The tree-based baselines showed smaller improvements over the corresponding Xie et al.^14,30^ random forest, ranging from 0.01 to 0.06 across the same three targets. Polymer diffusivity did not exceed the performance values reported by Xie et al.^14,30^ for any of our architectures, although the cross-validated standard deviation of 0.02 suggests that this shortfall reflects a systematic gap rather than partition variability. The improvements observed on three of four targets are driven primarily by the combination of chemically informed structural descriptors with the neural network architecture, rather than by the descriptors alone. This is consistent with the feature importance analysis discussed below, in which polymer flexibility and polarity metrics derived from SMILES emerged as the strongest predictors, descriptors that were not explicitly encoded in the fingerprint- and graph-based representations used in the referenced work.

### 3.2 Per-Model Behavior and Hyperparameter Sensitivity

Both the random forest and gradient boosting models were tuned through systematic manual search alongside Bayesian optimization using Optuna.^48^ For random forest, the Bayesian procedure iteratively sampled combinations of key parameters and evaluated each configuration on validation R^2^. Despite exploring many combinations across multiple trials, the optimized configurations did not surpass the manually tuned model, yielding validation scores below 0.5. Manual tuning was therefore used to identify the final configuration. We varied the number of estimators between 200 and 1500, maximum tree depth between 5 and 500, and minimum samples per leaf and per split between 5 and 30. These ranges were refined based on validation performance since extremely shallow trees underfit the data while excessively deep trees increased variance without improving predictive accuracy. The final random forest configuration used 800 estimators, maximum depth of 20, and minimum samples per leaf and per split of 5 and 10 respectively, with parallel processing enabled. This configuration was selected on the basis of consistent predictive stability across multiple random states and superior validation performance across target features. Gradient boosting was tuned across a comparable parameter range. Maximum depth was varied from 2 to 50, number of estimators from 200 to 1500, minimum samples per leaf and per split between 5 and 30, learning rate between 0.1 and 0.5, and maximum feature fraction between 0.2 and 0.9. The final gradient boosting configuration used 500 estimators, a learning rate of 0.1, maximum depth of 3, minimum samples per split of 10, minimum samples per leaf of 5, and a maximum feature fraction of 0.7. These configurations reflect a bias-variance balance in which shallow trees underfit the data while excessively deep trees increase variance without improving generalization. The neural network permitted a relatively high learning rate of 0.001 without overfitting, owing to the early stopping mechanism that halted training once validation performance plateaued. Architectural hyperparameters were varied over broad ranges. The number of hidden layers was varied from 1 to 3, neurons per layer from 64 to 256, batch sizes from 4 to 64, and early stopping patience from 10 to 40 epochs. These ranges were refined on the basis of validation R^2^. The final architecture consisted of two hidden layers of 128 neurons each with ReLU activations, trained using the Adam optimizer^45^ and mean squared error loss. A batch size of 16 produced the most stable convergence for conductivity, TFSI diffusivity, and polymer diffusivity. A smaller batch size of 8 improved gradient updates and predictive accuracy specifically for Li-ion diffusivity. Batch size was therefore selected on a per-target basis.

Model behavior varied meaningfully across targets. Random forest and gradient boosting produced particularly stable predictions for conductivity and polymer diffusivity, where transport is governed by a small number of dominant descriptors such as backbone flexibility and ionic content. Ensemble tree models exploit these dominant descriptors through hierarchical splitting. Because these relationships are relatively strong and monotonic, tree-based models were able to capture them without requiring extremely complex feature interactions. Diffusivity of lithium ions, by contrast, presented greater modeling difficulty. Li-ion transport depends not only on polymer mobility but also on transient coordination environments, ion hopping mechanisms, and local solvation structures. These effects produce subtler and more continuous nonlinear dependencies in the descriptor space. Gradient boosting improved lithium-ion diffusivity predictions relative to random forest, while the neural network offered a further advantage. The layered architecture of the neural network can represent continuous multivariable interactions rather than stepwise splits. This appears better suited to capturing coupled dependencies among polymer polarity, salt concentration, and backbone flexibility. Lithium diffusivity, for example, is not controlled by a single dominant feature but rather by the balance between coordination strength and segmental motion. Neural networks are better equipped to represent these multivariable dependencies.

The cross-validation results additionally clarified the practical advantage of the neural network over ensemble tree methods. While mean R^2^ differences between the neural network and the tree baselines were modest for conductivity (approximately 0.15) and Li-ion diffusivity (approximately 0.10), the difference in fold-to-fold variability was substantially larger. Standard deviations across the five folds were 2 to 3 times greater for the ensemble models than for the neural network on the three ion-transport targets. Inspection of the lowest-performing folds indicated that tree-based models occasionally failed on partitions containing chemistry clusters underrepresented in the training set, whereas the neural network’s smoother function class generalized to these regions with only modest performance loss. This robustness gap represents a stronger practical case for the neural network than the single-fold performance numbers alone would suggest, and it is most pronounced for the ion-transport targets where transport is governed by coupled multidescriptor dependencies rather than a small number of dominant features. To probe whether the tree-model instability on the lowest-performing folds arose from specific chemistry coverage gaps, we compared training and test distributions across all engineered features for the conductivity target. Univariate Kolmogorov–Smirnov tests indicated no statistically significant distributional shift on any feature in any fold, with all p-values exceeding 0.10. Sparse-element frequencies for fluorine, sulfur, chlorine, silicon, and phosphorus differed by no more than a few tenths of a percent between fold 4’s training and test partitions, and multivariate nearest-neighbor distances in standardized feature space were comparable across folds, ruling out both single-feature shift and joint-space extrapolation as explanations. No single dominant descriptor or chemistry subclass could therefore be identified as the source of the tree-model performance drop. This suggests that the ensemble sensitivity arises from cumulative small distributional differences distributed across many descriptors and their interactions, rather than from underrepresentation of a specific chemical family. This pattern is consistent with the known variance of tree-ensemble predictions under random partitioning of chemically heterogeneous data sets.^49,50^ The comparable robustness of the neural network across the same folds indicates that its smoother function class integrates over these small distributional differences more gracefully than the hierarchical partitions produced by decision trees.

These performance gains must be interpreted alongside the neural network’s greater sensitivity to training dynamics. Unlike ensemble models, which are relatively robust to data set size and noise, neural networks require careful tuning to avoid memorization of the training data. Early stopping was essential to prevent overfitting by halting training once validation performance plateaued. Batch size adjustments also materially altered convergence behavior, particularly for Li-ion diffusivity, where reducing batch size improved gradient updates and predictive accuracy. Ensemble methods therefore remained the more robust baseline across data set variations. They are particularly useful when working with consistent chemical descriptors and moderate data set sizes. The neural network extracted deeper interactions at the cost of additional regularization overhead. This complementary behavior highlights the value of using multiple model classes when studying structure–property relationships in amorphous polymer electrolytes.

### 3.3 Numerical Scaling of Diffusivity Targets

The three diffusivity targets take values on the order of 10^–8^, whereas conductivity spans a numerically well-conditioned range. Initial training on the raw diffusivity values produced flatlined predictions and R^2^ values below 0.3 for both random forest and gradient boosting. Although tree-based regressors are, in principle, invariant to linear rescaling of the target, this invariance holds only when the split-selection criterion can resolve differences between candidate partitions. At target magnitudes of ∼10^–8^, the squared-error terms used in split evaluation fall to ∼10^–16^, comparable in scale to the default tolerance thresholds governing whether a split is accepted (for example, the minimum impurity decrease required to expand a node) and to the precision floor of standard double-precision arithmetic. Under these conditions, differences in split quality become difficult to distinguish from numerical noise, and the models fail to identify informative partitions of the feature space. Uniform rescaling of the diffusivity targets by a factor of 10^8^ prior to training moved the loss into a well-conditioned numerical range and eliminated this pathology, significantly improving predictive accuracy across the ensemble models. Because the rescaling is a single global multiplicative constant, the ordering and relative magnitudes of targets are preserved exactly, and the learned structure–property relationships are unaffected. Conductivity targets were retained in their original scale, since their numerical range did not exhibit this instability. Uniform rescaling was preferred over normalization or standardization because the diffusivity values were already physically meaningful; multiplying by a constant improves numerical resolution without introducing data set-dependent parameters or obscuring the physical interpretation of the targets. The neural network was not affected by this issue, owing to input standardization and the magnitude adaptivity of gradient-based optimization with adaptive learning rates. This distinction highlights a disadvantage of gradient-based models on data sets involving closely spaced or small-magnitude targets and underscores the importance of numerical conditioning when applying tree-based machine learning to electrochemical transport data.

### 3.4 Feature Importance and Mechanistic Interpretation

Feature importance rankings were extracted for both ensemble models across all four targets (Figure 3), and SHAP attributions were computed for the neural network across the same targets (Figure 4). Several trends were consistent between random forest and gradient boosting, and the neural network SHAP analysis recovered the same dominant descriptors while additionally providing directional attribution and per-sample resolution. This convergence across three independently trained architectures with differing inductive biases indicates that the learned rankings reflect genuine structure–property relationships rather than model-specific artifacts. Broadly, descriptors describing polymer backbone flexibility, polarity, and ionic environment dominated predictions, while simple elemental composition descriptors contributed weakly. We interpret these rankings in terms of three coupled physical mechanisms known to govern ion transport in amorphous polymer electrolytes: segmental mobility of the polymer matrix, ion solvation and coordination, and charge carrier availability.

**Figure 3:**
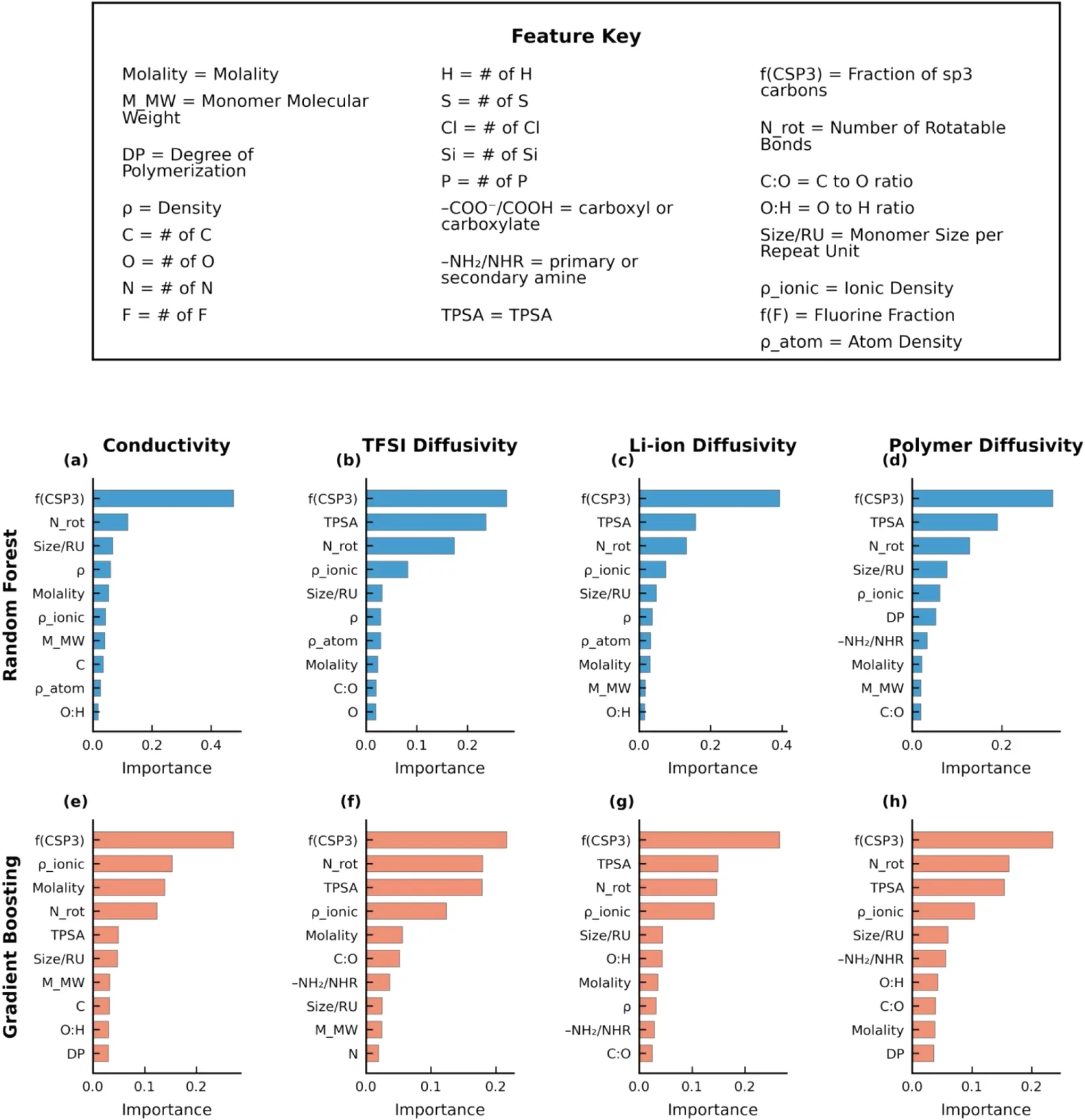
Feature importance rankings for the random forest model in the top row (panels a to d) and the gradient boosting model in the bottom row (panels e to h) across the four transport properties, showing the top 10 features per panel ordered from most important at the top to least important at the bottom, with feature abbreviations used throughout the panels defined in the key at the top of the figure. Differences in feature rankings between random forest and gradient boosting for the same target property reflect the distinct ways each algorithm partitions feature contributions, so features that appear consistently near the top across both models represent the most robust predictors of that property.

**Figure 4:**
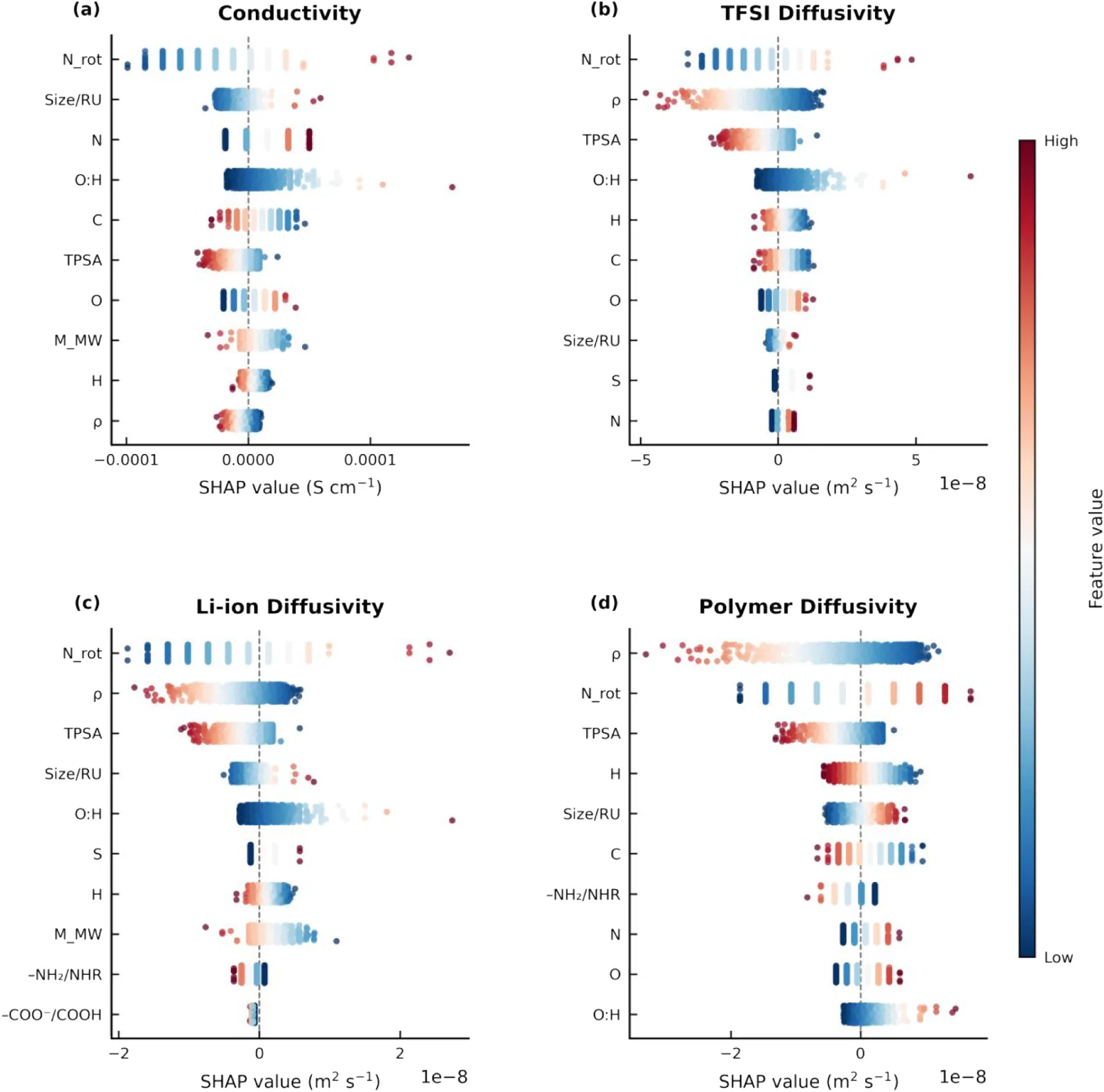
SHAP beeswarm summary for the neural network model across the four transport targets: (a) ionic conductivity, (b) TFSI diffusivity, (c) Li-ion diffusivity, and (d) polymer diffusivity. Each point represents a held-out test sample; horizontal position indicates the SHAP value in physical target units, and point color encodes the underlying feature value, normalized per feature between low (blue) and high (red). Features are ranked by mean absolute SHAP value, with the most influential feature shown at the top of each panel. Positive SHAP values indicate that the feature increased the model prediction relative to the expected value, and negative SHAP values indicate a decrease. Feature abbreviations are as defined in Figure 3.

Backbone flexibility descriptors emerged as the strongest predictors across all four targets. FractionCSP3, which measures the fraction of sp^3^-hybridized carbons in the polymer backbone, was the single most important feature in both ensemble models, and the number of rotatable bonds was the single most important feature in the neural network SHAP attribution for every target. Because sp^3^ carbons permit greater conformational freedom than sp^2^ carbons, FractionCSP3 acts as a direct proxy for chain flexibility. Higher saturation corresponds to backbones capable of greater segmental motion. Since ion transport in amorphous polymer electrolytes is strongly coupled to polymer chain flexibility, increased backbone flexibility facilitates ion hopping and enhances both conductivity and diffusivity. The consistent dominance of this feature across conductivity and all three diffusivity targets reinforces the central role of segmental mobility in governing transport behavior. The number of rotatable bonds ranked comparably high, reinforcing this interpretation. Freely rotating single bonds along the polymer chain increase configurational entropy and enable the local relaxations required for ion hopping. Monomer molecular weight and monomer size per repeat unit contributed similarly, reflecting how repeat unit geometry modulates chain packing density and the availability of transport pathways.

The three architectures nevertheless differed in how importance was distributed across the feature set. Both tree ensembles concentrated attribution on FractionCSP3 for every target, whereas SHAP did not rank FractionCSP3 among the top ten features for any target of the neural network. This discrepancy reflects two established properties of impurity-based importance: impurity scores are systematically inflated for continuous features that admit many candidate splits,^51^ and tree ensembles concentrate credit on the single feature providing the most efficient partition even when other features carry overlapping information. The neural network appears to encode the same backbone-character axis implicitly through combinations of rotatable bond count, atomic-composition descriptors, and monomer size, and SHAP distributes the attribution across these correlates rather than concentrating it on FractionCSP3 itself. The convergent mechanistic conclusion is therefore preserved across models even though the specific feature carrying the numerical attribution differs. It should be noted that SHAP attribution itself is not immune to distortion under correlated inputs, since Shapley values distribute credit across features whose marginal contributions overlap. The interpretation advanced here therefore rests not on the reliability of any single attribution method but on the convergence of three architectures with differing inductive biases onto the same underlying mechanistic axes of backbone flexibility, polarity, and ionic environment.

Polarity descriptors were the second-most influential category, with the strongest effect observed for TFSI and Li-ion diffusivity. TPSA, which quantifies the surface area contributed by polar atoms such as oxygen, ranked highly for both diffusivity targets. Higher TPSA reflects a greater density of coordination sites, promoting salt dissociation and stabilizing mobile ions. Excessive coordination, however, can reduce mobility by trapping Li^+^ ions. This descriptor therefore captures a physically nontrivial balance rather than a monotonic effect. Additional polarity indicators such as O/H and C/O ratios contributed at a lower but nonzero level, reflecting the importance of ether and hydroxyl functionalities in Li^+^ solvation. Functional group descriptors such as carboxyl/carboxylate and primary/secondary amine counts showed intermediate importance. While these groups can coordinate lithium ions, their presence does not universally improve transport. Strong binding can immobilize ions and reduce conductivity, which may explain their lower ranking relative to broader polarity metrics such as TPSA.

Because SHAP values retain the sign of each feature contribution, the neural network attribution supports directional mechanistic claims that impurity importance cannot provide. Rotatable bond count and the O/H ratio contributed positively to predicted transport across all four targets, consistent with greater backbone flexibility and higher ether-oxygen density enhancing both segmental relaxation and cation coordination site availability. Mass density and TPSA contributed negatively, consistent with a free-volume suppression of hopping and with strong polar coordination trapping mobile species at binding sites. A chemically resolved finding emerged specifically for Li-ion diffusivity: primary and secondary amine groups and carboxyl or carboxylate groups both contributed negatively to Li-ion mobility, and neither functional group appeared with comparable magnitude in the SHAP attributions for TFSI or polymer diffusivity. This target-specific behavior is mechanistically coherent since amines and carboxylates are hard Lewis-base donors that bind Li^+^ tightly without similarly interacting with the TFSI- anion or with polymer backbone motion. Both tree ensembles independently ranked *primary_or_secondary_amine* within the top ten features for Li-ion diffusivity, providing three-way robustness for this chemically specific claim.

Several structural and morphological descriptors demonstrated moderate predictive value. Degree of polymerization influences polymer and ion diffusivity, since longer chains increase entanglement and reduce overall mobility. Atom density and bulk density also contributed modestly, consistent with their interpretation as free-volume proxies. Lower packing density leaves more accessible volume for ion motion. At the bottom of the rankings were simple elemental fraction descriptors, including Cl, Si, P, F, S, and overall fluorine fraction, along with isolated elemental counts such as total carbon, hydrogen, and nitrogen. These descriptors act as coarse compositional indicators that carry little mechanistic information in isolation. They do not directly capture polymer dynamics, solvation environments, or salt dissociation behavior. Without structural context, an atomic count cannot distinguish, for example, a fluorine in an inert side chain from a fluorine in a coordinating environment. Their low importance is therefore consistent with the underlying physics rather than surprising. This suggests that ion transport is governed more by functional arrangement and polymer architecture than by raw elemental composition.

The collective feature rankings reinforce established physical models of amorphous polymer electrolytes, where ionic transport is controlled by three coupled mechanisms. The first is segmental mobility, enabled by flexible, saturated backbones and rotatable bonds. The second is ion solvation, determined by polymer polarity and coordination sites. The third is charge carrier availability, governed by ionic density and molality. High-importance features are directly mapped onto these mechanisms, explaining the strong predictive performance of ensemble models trained on chemically meaningful descriptors.

#### 3.4.1 Data Set and Feature Limitations

While the feature importance results match chemical expectations, several limitations of the data set and feature set constrain the interpretation. Several low-importance elemental descriptors, including Si, P, Cl, and F, may rank low not because they are chemically irrelevant but because they appear in only a small subset of the polymer data set. Sparse representation limits the model’s ability to associate these elements with transport behavior and can suppress their apparent importance. More fundamentally, most of the engineered features describe the static structure of the polymer but not its dynamics. Ion transport in amorphous polymer electrolytes depends strongly on polymer motion, including chain relaxation and segmental dynamics. Since features such as glass transition temperature and molecular motion data were not included, the models had to rely on indirect proxies such as FractionCSP3 and rotatable bond count to infer chain mobility. This likely places an upper bound on achievable predictive accuracy for the diffusivity targets. Additionally, several descriptors within our feature set are chemically overlapping. TPSA, oxygen content, and coordination group counts all relate to polarity, and their importance may be split among correlated features in ways that obscure the true contribution of any individual descriptor. The differing treatment of FractionCSP3 between impurity-based and SHAP-based attribution, discussed above, reflects this same underlying redundancy and highlights the value of using multiple attribution methods when interpreting chemical descriptors. While the models still captured major chemical trends, incorporating more diverse chemistries, adding dynamic transport descriptors, and applying correlation-aware feature selection could improve predictive accuracy in future work.

### 3.5 Implications for Machine Learning of Polymer Electrolytes

Taken together, the model comparison and feature importance analysis suggest several practical implications for future ML frameworks targeting polymer electrolytes. First, chemically informed structural descriptors derived from SMILES, when paired with a neural network architecture, substantially exceed prior benchmarks on three of four transport targets. The corresponding gains for tree-based baselines under identical descriptor sets were smaller, indicating that descriptor engineering and architectural capacity contribute jointly rather than independently to the observed improvements. This indicates that explicit encoding of flexibility and polarity provides predictive signal beyond generic fingerprint or graph representations. The feature importance results additionally provide useful guidance for descriptor selection. Backbone flexibility descriptors such as FractionCSP3 and rotatable bond count should be prioritized in any predictive model, since ion transport in amorphous systems is strongly coupled to polymer dynamics. Polarity-related features such as TPSA and oxygen coordination metrics are essential for lithium and anion diffusivity. Salt concentration descriptors, such as ionic density and molality, are critical for conductivity prediction. Second, ensemble tree-based methods and neural networks occupy complementary regimes. Random forest and gradient boosting offer stable, interpretable baselines particularly well suited to targets governed by a small number of dominant descriptors and moderate data set sizes with structured chemical features. The complementary attribution methods available for the two model families further support this pairing: impurity-based importance from ensemble models identifies dominant partition features, while SHAP attribution of the neural network provides directional and per-sample interpretability that isolates chemically specific effects such as the negative contribution of amine and carboxylate groups on Li-ion mobility. Future studies may therefore benefit from hybrid approaches that use ensemble models for baseline screening and neural networks for high-precision predictions in chemically diverse polymer electrolyte systems. Third, the consistent ranking of segmental-mobility and polarity descriptors across models and targets provides a compact guide for descriptor selection in future studies. Any predictive framework for amorphous polymer electrolytes should include, at minimum, descriptors capturing backbone flexibility, ion coordination environment, and carrier concentration. Physics-informed descriptors, uncertainty quantification, and expanded MD data sets remain natural directions for further improvement.

## 4 Conclusions

In this study, we applied machine learning to predict four key transport properties in amorphous polymer electrolytes. These included ionic conductivity, TFSI diffusivity, Li-ion diffusivity, and polymer diffusivity. We used an MD data set of more than 6,000 polymer electrolyte systems enhanced with engineered structural and chemical descriptors. Three modeling frameworks were trained and compared, namely random forest, gradient boosting, and neural networks. These models were evaluated under 5-fold cross-validation. All three models successfully captured structure–property relationships. Ensemble tree methods provided stable baseline performance. The neural network achieved the highest overall accuracy across most targets. For conductivity, TFSI diffusivity, and Li-ion diffusivity, our neural network exceeded the benchmarks previously reported by Xie et al.^14,30^ on the same data set by approximately 0.10 to 0.18 in cross-validated R^2^. Ensemble tree baselines showed smaller improvements of 0.01 to 0.06. Beyond mean performance, the neural network exhibited substantially greater robustness across data partitions. Fold-to-fold standard deviations were two to three times smaller than those of the tree ensembles for the three ion-transport targets. Polymer diffusivity predictions were consistent across model types and remarkably stable across folds, with standard deviations at or below 0.02. However, they did not surpass the highest value reported by Xie et al.^14,30^ Across all models and targets, typical prediction errors fell within a factor of 1.4 of the MD reference values (*log*_10_ *MAE* of 0.100 to 0.135). Feature importance analysis revealed that polymer backbone flexibility, polarity, and ionic environment were the dominant factors governing transport behavior. Descriptors such as FractionCSP3, rotatable bonds, TPSA, and ionic density consistently ranked among the most influential. These rankings reinforce established physical models linking segmental motion, ion solvation, and charge carrier availability to electrolyte performance. Directional SHAP attribution on the neural network additionally identified primary and secondary amine groups and carboxylate groups as specific negative contributors to lithium-ion mobility. This is a target-specific finding that impurity-based importance alone could not resolve. These findings validate the chemical relevance of the engineered feature set. They also highlight the importance of mobility and coordination-focused descriptors in predictive modeling. The improvements observed over prior benchmarks were driven primarily by the neural network’s exploitation of the engineered descriptor set. This suggests that explicit encoding of chemically meaningful descriptors provides predictive signal beyond generic fingerprint or graph representations when combined with sufficient model capacity. Overall, this work illustrates how machine learning can accelerate the screening and design of polymer electrolytes while retaining interpretability of the underlying structure–property relationships. Continued expansion of data sets, incorporation of dynamic transport descriptors such as glass transition temperature and segmental relaxation times, and integration of physics-informed constraints and uncertainty quantification represent natural directions for further improvement. These could support the development of next-generation polymer electrolytes for lithium batteries.

## #

Department of Electrical and Computer Engineering, Rutgers University, New Brunswick, NJ 08901
